# CA-125, CA-153, and CYFRA21-1 as clinical indicators in male lung cancer with ocular metastasis

**DOI:** 10.7150/jca.36238

**Published:** 2020-02-20

**Authors:** Biao Li, Qing Yuan, Yu-Ting Zou, Ting Su, Qi Lin, Yu-Qing Zhang, Wen-Qing Shi, Rong-Bin Liang, Qian-Min Ge, Qiu-Yu Li, Yi Shao

**Affiliations:** Department of Ophthalmology, The First Affiliated Hospital of Nanchang University, Jiangxi Province Ocular Disease Clinical Research Center, Nanchang 330006, Jiangxi, People's Republic of China

**Keywords:** ocular metastasis, lung cancer, men, potential indicators, risk factors, smoking preference

## Abstract

Despite recent improvements in diagnosis and therapy, lung cancer remains the most common malignant tumor in males, with high morbidity and mortality. As the annual incidence continues to increase worldwide, the prognosis for male patients with lung cancer remains unsatisfactory. Interestingly, smoking is associated with lung cancer and ocular lesions by altering risk factors such as carbohydrate antigen (CA)-125, CA-153 and cytokeratin-19 fragment (CYFRA21-1). A diagnostic standard for serum biomarker levels of ocular metastasis (OM) in males with lung cancer is therefore urgently needed. In this retrospective analysis, we examined the relationship between smoking preference and OM in male patients with lung cancer to identify an independent prognostic factor or establish a quantitative indicated standard for OM using the clinical indexes from 2238 cases of male lung cancer. The combination of CA-125, CA-153 and CYFRA21-1 could help diagnose OM in male lung cancer patients. This finding might lead to more timely diagnosis and effective therapies.

## Introduction

Lung cancer is one of the most malignant tumors in the world, leading to the highest morbidity and mortality among male cancer patients 1. Patients in low- and middle-income countries now account for more than 50% of lung cancer deaths annually 1 since they have high smoking rates. Lemjabbar-Alaoui and colleagues reported that about ~90% of lung cancer is caused by smoking and tobacco product use 2. Multiple inherited 3 and acquired mechanisms of susceptibility to lung cancer have also been proposed 2.

Smoking is one of the most significant pathogenic factors of male lung cancer^4^, ^5^. This habit is also associated with eye diseases like hyperopia, delayed corneal epithelial healing, progression of Fuchs' endothelial corneal dystrophy, age-related nuclear cataract, retinal diseases, uveitis, optic neuropathies, and thyroid-associated orbitopathy 6. We therefore suspected that smoking might be associated with the occurrence of ocular metastasis (OM) from lung cancer.

Rapid metastasis significantly contributes to the high mortality rates of lung adenocarcinoma 7, 8. One group described a rare case of primary lung adenocarcinoma with concomitant gastric, duodenal, bone, and mediastinal lymph node metastases, even gastrointestinal metastases 8. The eye is a rare metastasis site for lung cancer 9 because of vasculature 10 and lymphatics 11. The incidence of OM from metastatic lung cancer was reported as 6.7% 12. Traditional computed tomography (CT) and magnetic resonance imaging (MRI) cannot diagnose early stage lung cancer. Accurate diagnosis is needed to guide treatments including surgery, radiation, chemotherapy, and targeted therapies 2.

Namad and colleagues reported a case of bilateral choroidal metastasis from non-small cell lung cancer. A 59-year-old Caucasian female patient was diagnosed with malignant lung cancer, and during follow-up, she presented with bulge on her left eye. Her follow-up chest scan simultaneously showed increases in the size of lung nodules. Her performance status remained stable at that time, except for a mild increase in dyspnea 12. This case reflects the importance of early diagnosis of metastasis from lung cancer.

Compared with candidate lung cancer biomarkers using molecular diagnostics 13, 14, blood test is a simple diagnostic tool popular throughout China's hospitals. An effective blood test gives prognostic information to help clinicians make case judgements. Serum biomarkers have long been studied to establish a reliable standard to quickly identify metastasis, supplemented with “gold standard” diagnostic methods such as imaging and tissue biopsies.

In our retrospective analysis, we examined individual and composite serum indicators associated with OM in male lung cancer, and a diagnosis standard was developed.

## Materials and Methods

### Study design

This study was conducted in accordance with the Declaration of Helsinki and approved by the medical research ethics committee of the First Affiliated Hospital of Nanchang University. The methods implemented in this study were conducted under relevant regulations. Therefore, the study results are uncontested by all authors. Serum samples of male patients with lung cancer were preoperatively collected from July 5, 2005 to July 25, 2016. We looked at records from 2238 male lung cancer patients admitted to our hospital. Based on the histopathological examination of samples collected by surgical resection or needle biopsy techniques, patients were categorized into two groups: OM and non-OM (NOM). The inclusion criteria for the OM group were non-primary ocular malignant tumor, non-ocular benign tumor, non-hereditary disease, non-distant metastases (apart from the eyes), and non-cardiovascular or non-cerebrovascular disease. OM diagnoses were made using CT or MRI and proved histologically or cytologically. The inclusion criteria for the NOM group were no secondary lung cancers, no organ metastases, and no lymph node metastases. All subjects of the study were fully informed of the purpose of the clinical study, agreed to participate, and signed informed consent forms.

### Data collection

All male patients who had pulmonary carcinoma proved by imaging examination such as CT and MRI were recruited for this study. Diagnoses were confirmed by pulmonary tissue biopsy. The clinical data of this retrospective study including age, histopathology types, and treatments were collected from the records in the medical system. Serum levels of metastatic general serum biomarkers, such as alpha-fetoprotein (AFP), carcinoembryonic antigen (CEA), carbohydrate antigen (CA)-125, CA-199, CA-153, CA-724, cytokeratin-19 fragment (CYFRA21-1), total prostate-specific antigen (TPSA), free prostate-specific antigen (FPSA), and neuron-specific enolase (NSE) as well as alkaline phosphatase (ALP), calcium, and hemoglobin (HB) were all recorded at the time of initial diagnosis of lung cancer.

### Statistical analyses

Student's t tests and chi square tests were performed to identify differences between the OM and NOM groups. Then, binary logistic regression models were generated to assess the values of different serum biomarkers for OM in male lung cancer. Receiver operating characteristic (ROC) curves were plotted, and the areas under the curve (AUCs) were calculated to determine the diagnostic utilities of risk factors. P < 0.05 for a two-sided test was considered statistically significant. Statistical analyses were performed with SPSS version 17.0 software (SPSS Inc, Chicago, IL, USA), MedCalc18.6.0 statistical software (MedCalc, Ostend, Belgium) and Excel 2016 software (Microsoft Corp, Redmond, WA, USA). Clinical measurement data are presented as means ± standard deviations (SD).

## Results

### Demographics and clinical characteristics

A total of 2238 male patients including 50 OM cases (16 orbital metastasis and 34 intraocular metastasis cases) and 2188 NOM cases were recruited for this study. The average ages of the OM and NOM subjects were 58.82 ± 10.32 and 61.23 ± 10.32 years, respectively (P > 0.05). Smoking preference was significantly different between groups (P < 0.05) which confirmed that smoking is associated with OM in male lung cancer patients and is related to the occurrence of lung adenocarcinoma. We also observed differences histopathology types between the two groups (P < 0.05). Squamous cell carcinoma and adenocarcinoma were the most common types in the NOM and OM groups, respectively. From 2005 to 2016, the majority of patients underwent chemotherapy and surgery. Patients' detailed clinical features are listed in Table 1. Table 2 shows the relationship between smoking and histopathology types.

### Differences in the clinical features and risk factors for OM

Compared with the NOM group, significantly higher concentrations of AFP, CEA, CA-125, CA-199, CA-153, CYFRA21-1, ALP, and TPSA were measured in the OM group (P < 0.05). The serum levels of CA-724, FPSA, NSE, calcium, and HB were not significantly different between the two groups (P > 0.05). The results are shown in Table 3. The binary logistic regression results showed that CA-125, CA-153, and CYFRA21-1 were independent risk factors for OM. The detailed results are listed in Table 4.

### The cut-off values, AUCs, sensitivities, and specificities of CA-125, CA-153, and CYFRA21-1 for diagnosing OM

**Figure 1:**
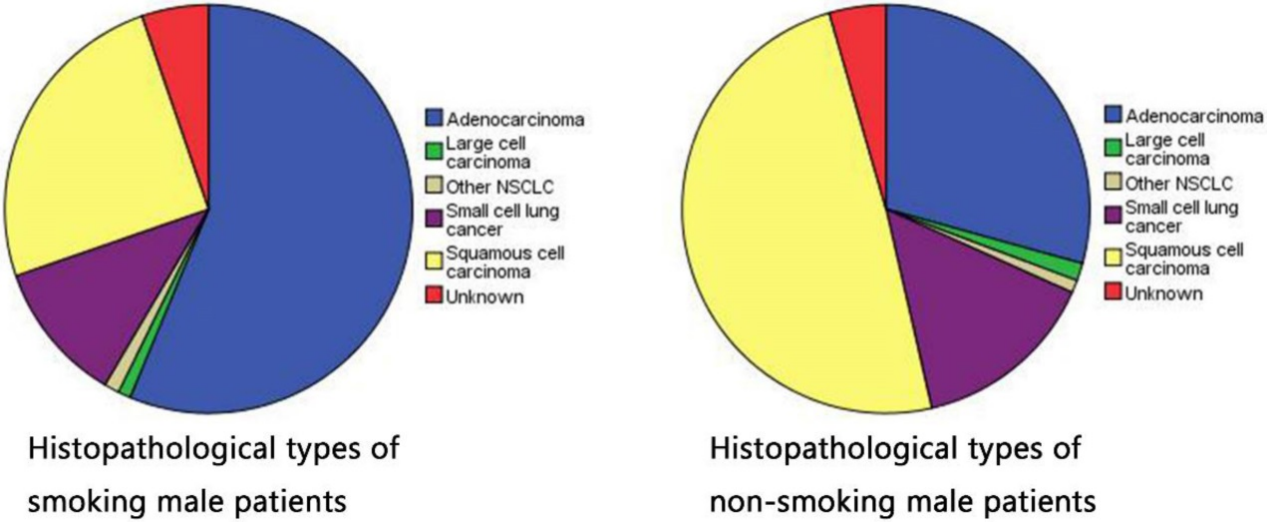
Histopathological types of smoking and non-smoking patients in male lung cancer.

**Figure 2:**
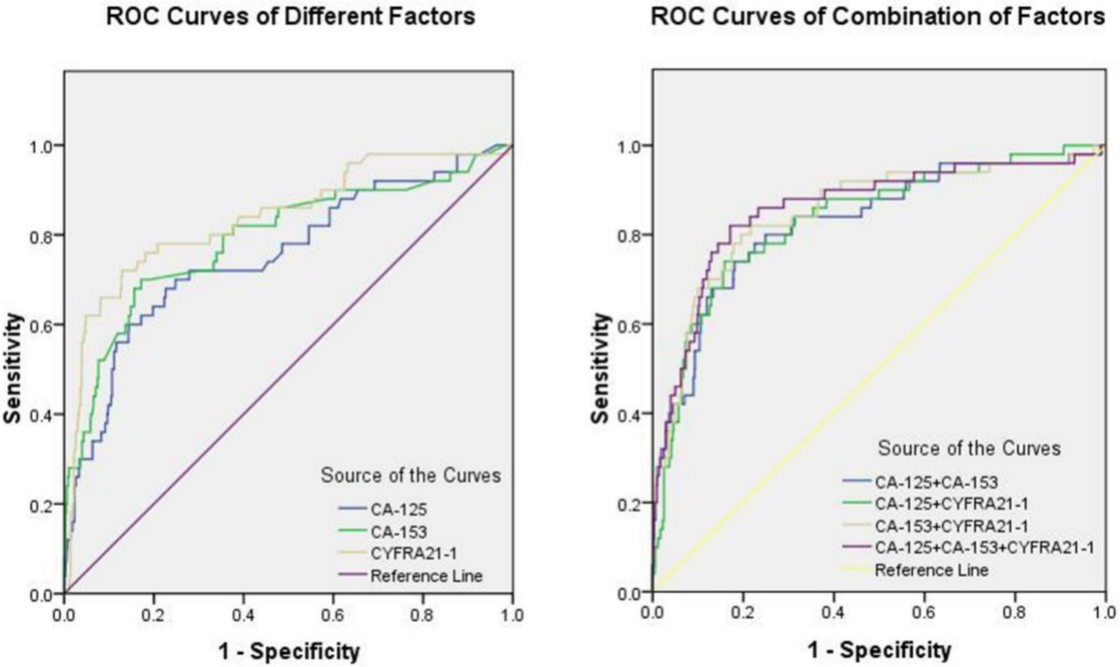
The ROC curves of risk factors for detecting OM in male lung cancer. **Notes:** (a) ROC curves of CA-125, CA-153 and CYFRA21-1 as single risk factor of OM. (b) ROC curves of combination of CA-125, CA-153 and CYFRA21-1 to detected OM in male lung cancer. **Abbreviations:** ROC, receiver operating characteristic; OM, ocular metastasis.

Fig. 1 shows the ROC curves for CA-125, CA-153, and CYFRA21-1 as male-specific single risk factors, and Table 5 shows the cut-off values of CA-125, CA-153, and CYFRA21-1 as 76.56 U/ml, 22.33 U/ml, and 10.70 ng/ml, respectively. The AUC of CYFRA21-1 was the highest among single risk factors. Fig. 2 shows the ROC curves of combinations of serum levels for CA-125 + CA-153, CA-125 + CYFRA21-1, CA-153 + CYFRA21-1, and CA-125 + CA-153 + CYFRA21-1. We found that the combination of CA-125 + CA-153 + CYFRA21-1 had the largest AUC, which reached 0.859. Among all potential indicators, the combination of CA-125 + CA-153 + CYFRA21-1 had the highest sensitivity, while CYFRA21-1 had the highest specificity. All the results were statistically significant.

## Discussion

The incidence of lung cancer in males continually increased through the late 90s, although it has slightly declined 15. Lung cancer is still a serious malignant tumor. The incidence and mortality both seem to increase with age 16, and for people ≥65 it accounts for approximately 50% of all cancer cases and cancer-caused deaths 15. Despite extensive research and considerable medical progress, a cure for lung cancer remains elusive.

The main cause of lung cancer is certainly smoking 17, and it is highly possible that individual lifestyle and nutrition; genetic predisposition; and exposure to asbestos, arsenic, aromatic hydrocarbons, and pollution are also responsible for lung cancer cases 15.

Men who smoke have an increased risk of lung cancer. Numerous clinical investigations for pathological changes show that smoking is inextricably linked to lung disease. Smoking-related lung abnormalities are now an increasing public health concern 18. According to large-cohort studies, approximately 8% of smokers have interstitial lung abnormalities 19, and a variety of pathological and physiological abnormalities exist in the lungs of smokers and ex-smokers, including emphysema and various interstitial lung diseases (ILDs). Traditional smoking-related lung diseases include Langerhans cell histiocytosis (LCH), respiratory bronchiolitis-ILD (RB-ILD), and desquamative interstitial pneumonia (DIP) 20. Smoking is also a risk factor for chronic fibrosing interstitial pneumonias including usual interstitial pneumonia (UIP) and non-specific interstitial pneumonia (NSIP) 21, as well as unclassifiable idiopathic interstitial pneumonias (unclassifiable IIPs) 22. These abnormalities are associated with a relatively high risk of all-cause mortality 9, and some progress to pulmonary fibrosis 23. Finally, tuberculosis and pneumonia can also increase lung cancer risk 24.

At present, there is no worldwide unified diagnostic standard for tobacco dependence 25, 26. Generally, drug-dependent diagnostic standards including nicotine in the International Classification of Diseases (ICD-10) are used. A person can be considered to have a smoking preference if he has experienced or demonstrated at least 3 of the following 6 items in the past year: 1) strong craving to smoke; 2) difficulty controlling smoking behavior; 3) withdrawal symptoms sometimes occur when stopping or reducing the amount of smoking; 4) tobacco tolerance performance, that is, the need to increase the amount of smoking to obtain the experience that could be obtained by smoking less in the past; 5) gave up or reduced other activities and preferences in order to smoke; 6) and continued to smoke regardless of the dangers. According to the quantitative criteria of male smoking preference [Table 6], we classified moderate tobacco dependence and severe tobacco dependence into “Ever/Current” smoking statue, then classified mild tobacco dependence into “Never” smoking statue, finally divided all samples regardless of the presence of OM into two groups to observe their histopathological type. The smoking group has a higher probability of adenocarcinoma (56.3%), while the non-smoking group has a high incidence rate of squamous cell carcinoma (49.1%). Table 2 shows that smoking is more likely to cause lung adenocarcinoma. Yao et al. reported that smoking led to downregulation of histone deacetylase-2 and interleukin-8 and upregulation of tumor necrosis factor-α in lung adenocarcinoma tissues, and these changes were especially pronounced in smoking combined with chronic obstructive pulmonary disease 17.

Among malignant diseases, lung cancer is the leading cause of morbidity and mortality in China 27. Approximately 57% of lung cancer patients have distant metastases at the initial diagnosis, which is associated with poor outcomes 27. The incidence of OM from lung cancer is reported to be 0.1-7%, with adenocarcinoma and small cell lung cancer accounting for the highest proportions of these cases 28. Guo et al. reported an interesting case in which a woman presented with a 1-week history of left eye pain and blurred vision. Examination revealed central lung cancer in the right lower lobe with OM. After surgery and chemotherapy, her eye symptoms disappeared, the ocular lesion was well controlled without any specific ocular treatment, and she achieved prolonged progression-free survival (PFS) 28. If a quantitative indicator standard of serum risk factors for early diagnosis is established, it can effectively prolong PFS, which is one of the most significant goals of our study. Nanoparticle-based therapeutics are currently paving a new way for the diagnosis, imaging, screening, and treatment of primary and metastatic tumors 29. Eventually, treatment will be much easier, and the mortality rate will be greatly reduced.

Our results also identified AFP, CEA, CA-125, CA-153, CYFRA21-1, and TPSA as six clinically available biomarkers for OM in male lung cancer patients. CA-125, CA-153, and CYFRA21-1 are closely related to OM (P < 0.05). Meanwhile, AFP has been associated with liver cancer 30, and CEA is a broad-spectrum tumor marker used to assess the progression and prognosis of colorectal cancer 31, breast cancer 32, and lung cancer 33. Its specificity and sensitivity are limited, and CEA only has auxiliary value in diagnosis. Other studies revealed that serum CEA levels have a clear relationship with colorectal cancer stage 34, and TPSA is closely associated with prostate cancer 35.

Finally, we performed binary logistic regression analyses to assess the utility of CA-125, CA-153, and CYFRA21-1 as serum biomarkers for OM in male lung cancer. The highest sensitivity and specificity for a single risk factor were for CYFRA21-1, at 72.00% and 87.09%, respectively, while the values for the combination of CA-125, CA-153, and CYFRA21-1 were 82.00% and 82.92%, respectively. The combined value for the diagnostic specificity of the three risk factors was greater than any alone. The ROC curves show that the combination of CA-125 + CA-153 + CYFRA21-1 had the highest AUC value (0.859) for the diagnosis of OM in male lung cancer. Accordingly, it could be a useful combination of biomarkers for early clinical detection of male lung cancer. A reliable, sensible, and convenient clinical standard can be provided using the cut-off values of CA-125 (> 76.56 U/ml), CA-153 (> 22.33 U/ml), and CYFRA21-1 (> 10.70 ng/ml). A three-biomarker panel (CA-125, CA-153, and CYFRA21-1) could allow classification of lung cancer with or without OM, with excellent sensitivity and specificity.

There are some limitations inherent to our study. First, the sample size of this retrospective analysis is small, especially for the OM group, so it can only be determined that smoking affects these biomarker levels but is not fully identifiable as a key factor. Serum biomarkers were collected and analyzed at the time of diagnosis, not at baseline. The biomarkers developed thus far are not the most ideal due to the limited sensitivity and specificity both individually and as a panel. Finally, all male patients were diagnosed in the same hospital. Further studies are needed in large samples of patients from multiple centers.

In conclusion, the combination of CA-125 + CA-153 + CYFRA21-1 is of value in the diagnosis of OM in male lung cancer. If the patient's serum levels are CA-125 > 76.56 U/ml, CA-153 > 22.33 U/ml, and CYFRA21-1 > 10.70 ng/ml, CT or MRI should be performed to check for OM. The combination of CA-125, CA-153, and CYFRA21-1 level is also prognostically valuable. The higher the combined level, the higher probability of OM, which can be a supplementary diagnostic indicator for OM from lung cancer in male patients 36. And Table 7 shows some risk factors of metastases of male lung cancer in recent years.

## Figures and Tables

**Table 1:** The clinical characteristics of male patients with lung cancer

Characteristics	OM group		NOM group	P value^*^
	(n=50)		(n=2188)	
**Age^#^**				
Mean	58.82±10.32		61.23±10.32	0.157
**Smoking statue**				
Ever/Current	25(50.0%)		483(22.1%)	<0.001
Never	25(50.0%)		1705(77.9%)	
**Histopathological type^##^**				
Squamous cell carcinoma	7(14.0%)		970(44.3%)	<0.001
Adenocarcinoma	34(68.0%)		759(34.7%)	
Large cell carcinoma	0(0.0%)		29(1.3%)	
Small cell lung cancer (SCLC)	5(10.0%)		306(14.0%)	
Other NSCLC	0(0.0%)		23(1.1%)	
Unknown	4(8.0%)		101(4.6%)	
**Treatment**				
Surgery	7		555	
Chemotherapy	28		986	
Radiotherapy	12		130	
Symptomatic treatment	3		403	
Others	0		114	

**Notes:** OM group included 16 orbital metastasis cases and 34 intraocular metastasis cases. #: Student-t test was used. ##: Chi-square test was used. *: comparison between OM group and NOM group. P<0.05 represented statistically significant. **Abbreviations:** OM, ocular metastasis; NOM, non-ocular metastasis; NSCLC, non-small cell lung cancer.

**Table 2:** The histopathological type of male lung cancer with smoking or non-smoking

Histopathological type^#^	Smoking	Non-smoking	P value^*^
	(n=508)	(n=1730)	
**Squamous cell carcinoma**	127(25.0%)	850(49.1%)	<0.001
**Adenocarcinoma**	286(56.3%)	507(29.3%)	
**Large cell carcinoma**	5(1.0%)	24(1.4%)	
**Small cell lung cancer (SCLC)**	57(11.2%)	254(14.7%)	
**Other NSCLC**	6(1.2%)	17(1.0%)	
**Unknown**	27(5.3%)	78(4.5%)	

**Notes:** #: Chi-square test was used. *: comparison of all histopathological types between OM group and NOM group. P<0.05 represented statistically significant. **Abbreviations:** NSCLC, non-small cell lung cancer.

**Table 3:** Differences of tumor markers between male lung cancer patients with and without OM

Tumor markers	OM group	NOM group	t	P value
**AFP (ng/ml)**	2.72±1.47	1.73±1.55	-4.662	<0.001
**CEA (ng/ml)**	207.15±518.34	38.93±221.65	-5.057	<0.001
**CA-125 (U/ml)**	310.24±504.79	61.23±157.43	-10.060	<0.001
**CA-199 (U/ml)**	142.39±338.01	33.95±228.20	-3.280	0.001
**CA-153 (U/ml)**	96.86±127.80	18.64±27.86	-16.306	<0.001
**CA-724(U/ml)**	11.43±32.91	12.97±46.83	-0.347	0.737
**CYFRA21-1(ng/ml)**	31.94±23.57	8.72±27.41	-5.940	<0.001
**TPSA**	3.85±2.41	1.63±4.09	-6.254	<0.001
**FPSA**	0.31±0.19	2.30±26.48	1.121	0.264
**NSE (μg/L)**	34.20±33.94	25.99±40.87	-1.667	0.101
**ALP (U/L)**	116.64±62.72	91.74±66.29	-2.772	0.008
**Calcium (mmol/L)**	2.27±0.22	2.29±1.70	0.386	0.700
**HB (g/L)**	115.86±23.16	120.90±19.35	1.812	0.070

**Notes:** Independent samples-t test was applied. P<0.05 represented statistically significant. **Abbreviations:** OM, ocular metastases; NOM, non-ocular metastases; HB, hemoglobin.

**Table 4:** Risk factors of OM in male lung cancer patients

Factors	B	Exp(B)	OR (95% CI)	P
**AFP**	0.149	1.161	1.032-1.306	0.013
**CEA**	0.001	1.001	1.000-1.001	0.001
**CA-125**	0.002	1.002	1.002-1.003	<0.001
**CA-153**	0.015	1.015	1.011-1.019	<0.001
**CYFRA21-1**	0.009	1.009	1.005-1.013	<0.001
**TPSA**	0.037	1.038	1.012-1.064	0.003
**CA-199**	<0.001	1.000	1.000-1.001	0.040
**ALP**	0.003	1.000	1.000-1.005	0.018

**Notes:** Binary logistic Analysis was applied. P <0.05 represented statistically significant. **Abbreviations:** B, coefficient of regression; OR, odds ratio; CI, confidence interval; OM, ocular metastases; Exp(B), index of B coefficient; AFP, alpha-fetoprotein; CA, carbohydrate antigen; CYFRA, cytokeratin-19 fragment; TPSA, total prostate-specific antigen.

**Table 5:** The cutoff value, sensitivity, specificity and AUC for single risk factor in predicting OM in male lung cancer patients

Factor	Cut-off value	Sensitivity (%)	Specificity (%)	AUC	P
CA-125 (U/ml)	76.56	60.00	85.56	0.754	<0.001
CA-153 (U/ml)	22.33	70.00	82.76	0.790	<0.001
CYFRA21-1(ng/ml)	10.70	72.00	87.09	0.838	<0.001
CA-125+CA-153	-	74.00	81.89	0.827	<0.001
CA-125+CYFRA21-1	-	74.00	84.12	0.834	<0.001
CA-153+CYFRA21-1	-	80.00	80.45	0.850	<0.001
CA-125+CA-153+CYFRA21-1	-	82.00	82.92	0.859	<0.001

**Notes:** Sensitivity and specificity were obtained at the point of cutoff value. P <0.05 represented statistically significant. **Abbreviations:** AUC, area under the curve; CI, confidence interval; OM, ocular metastasis.

**Table 6:** Fagerstrm Tobacco Dependence Assessment Scale: Assessing Tobacco Dependence

Evaluation items	0	1	2	3
How long do you take the first cigarette after waking up in the morning?	>60 minutes	31-60 minutes	6-30 minutes	≤5 minutes
Are you having difficulty controlling smoking in many non-smoking areas?	no			yes
Which cigarette do you think you are most reluctant to give up?	other time			first in the morning
How many cigarettes do you smoke every day?	≤10	11-20	21-30	>30
Did you smoke more than the other hour in the first hour after waking up in the morning?	no			yes
Are you still smoking when you are sick in bed?	no			yes

**Note:** 0 to 3 points, for mild tobacco dependence; 4 to 6 points, for moderate tobacco dependence; ≥ 7 points, for severe tobacco dependence.

**Table 7:** The risk factors of metastases of male lung cancer

Author	Year	Histopathological type	Metastatic sites	Risk factor
Lina Wu et al 37	2017	NSCLC	Lymph node	MicroRNA-422a
Chu Y et al 38	2017	Adenocarcinoma	Lymph node	CLSTN1, CLU, NGAL
Brody R et al 39	2017	NSCLC	NS	PD-L1
Wu S et al 40	2016	NSCLC	Lymph node	B7-H3
Chen Y et al 41	2015	NSCLC	Brain	NSE
Jain L et al 42	2009	NS	NS	SNP
Oshiro Y et al^43^	2004	Adenocarcinoma	Liver	AFP
Nikliński J et al 44	1992	NSCLC	Lymph node	SCC
Hirashima T et al^45^	2000	NSCLC	NS	telomere
Pollán M et al^46^	2003	NSCLC	NS	CA-125
Zhou Y et al^47^	2017	NS	Bone	CA-125, ALP
Cedrés S et al 48	2011	NSCLC	Brain	CEA, CYFRA21-1,
Dan Liu et al 49	2017	Adenocarcinoma	Brain, lymph node	CA-125
Chen F et al^50^	2015	NS	Lymph node	CYFRA21-1, CEA
Lee DS et al^51^	2012	NSCLC	Brain	CEA
Morita S et al^52^	2019	NSCLC	Intertrabecular Vertebral	CEA
Shetty D et al^53^	2016	NSCLC	Thyroid gland, lymph node	PSMA

**Abbreviations:** NS, not specific; NSCLC, non-small cell lung cancer; SCC, squamous cell carcinoma antigen; CLSTN1, calsyntenin-1; CLU, clusterin; NGAL, neutrophil gelatinase-associated lipocalin; SNP, single nucleotide polymorphisms; PD-L1, programmed cell death-1; B7-H3, B7 homolog 3; PSMA, prostate-specific membrane antigen.
